# Considerations for the Treatment Strategy of Relapse After Tofacitinib Therapy in Alopecia Areata

**DOI:** 10.1111/jocd.70234

**Published:** 2025-07-02

**Authors:** Longyan Yao, Jiumei He, Na Lan, Yongmei Lv

**Affiliations:** ^1^ The Second Affiliated Hospital of Anhui Medical University Hefei China

**Keywords:** alopecia areata, JAK inhibitor, therapy, tofacitinib

## Abstract

**Background:**

Alopecia areata (AA) is an autoimmune disorder mediated by T cells, resulting in hair loss on the scalp, eyebrows, and body. Conventional treatments for AA often exhibit high recurrence rates and various side effects. Recently, Janus kinase (JAK) inhibitors have emerged as promising therapeutic options for managing AA and several other autoimmune disorders.

**Case Presentation:**

We present the case of a 26‐year‐old patient who initially responded well to Tofacitinib but subsequently experienced relapses during the treatment period. Corticosteroid therapy was effective in managing these relapses, leading to a transition to Ritlecitinib when Tofacitinib monotherapy became unsustainable.

**Discussion:**

In cases where monotherapy proves ineffective, alternative strategies such as combination therapy, dose optimization, or switching to different therapeutic agents should be considered. While the JAK–STAT signaling pathway plays a pivotal role in the pathogenesis of AA, it is likely that additional mechanisms also contribute to its development.

**Conclusion:**

We present a case report documenting secondary failure of tofacitinib in the treatment of AA. This case highlights potential insights into the pathogenesis of AA and may inform the development of future therapeutic strategies.

## 1 Introduction

Alopecia areata (AA) is a chronic autoimmune disorder characterized by patchy or extensive hair loss, significantly impacting psychosocial well‐being and quality of life [1]. With a global prevalence of 0.1%–0.2% and a lifetime risk of approximately 2%, AA affects individuals across all demographics but is uncommon in children under 3 years of age [2]. The pathogenesis of AA involves genetic predisposition, immune dysregulation (particularly CD8+ T‐cell‐mediated attacks on hair follicles), and oxidative stress that disrupts immune privilege [1].

Traditional therapies such as corticosteroids and immunosuppressants have limited efficacy and raise safety concerns [1, 3, 4]. Recent advances highlight JAK inhibitors, particularly tofacitinib (a selective JAK1/3 inhibitor), as promising therapeutic agents. Studies demonstrate that 69.1% of patients achieve more than a 50% improvement in SALT scores, with 44.3% attaining over 90% hair regrowth [5]. However, relapse remains a significant challenge: 58% of patients experience notable fluctuations in SALT scores within 18 months [6], and 8 out of 22 patients in one study experienced recurrence despite continuous therapy [7]. Clinically observed secondary failure, defined as an initial response followed by a loss of efficacy, underscores the need for personalized treatment strategies and combination approaches to sustain remission. Ongoing research focuses on optimizing JAK inhibitor regimens while balancing safety and the durability of the therapeutic response.

## 2 Case Report

A 26‐year‐old female patient presented with patchy hair loss affecting her scalp, eyebrows, and pubic area, which began 9 years ago. Her hair growth was notably delayed, significantly impacting her appearance and causing considerable distress in daily life. Prior to this, she had no other physical abnormalities, nail changes, or history of similar conditions, and there was no family history of such diseases. She had previously tried oral danazol, sulfasalazine enteric‐coated tablets, triamcinolone acetonide tablets, topical clobetasol propionate cream, and minoxidil. While these treatments led to partial recovery of her eyebrows and pubic hair, her scalp hair did not improve and continued to worsen. Intralesional corticosteroid therapy was subsequently attempted but yielded unsatisfactory results, leading to the discontinuation of medication. Throughout this period, there was no significant regrowth of scalp hair.

**FIGURE 1:**
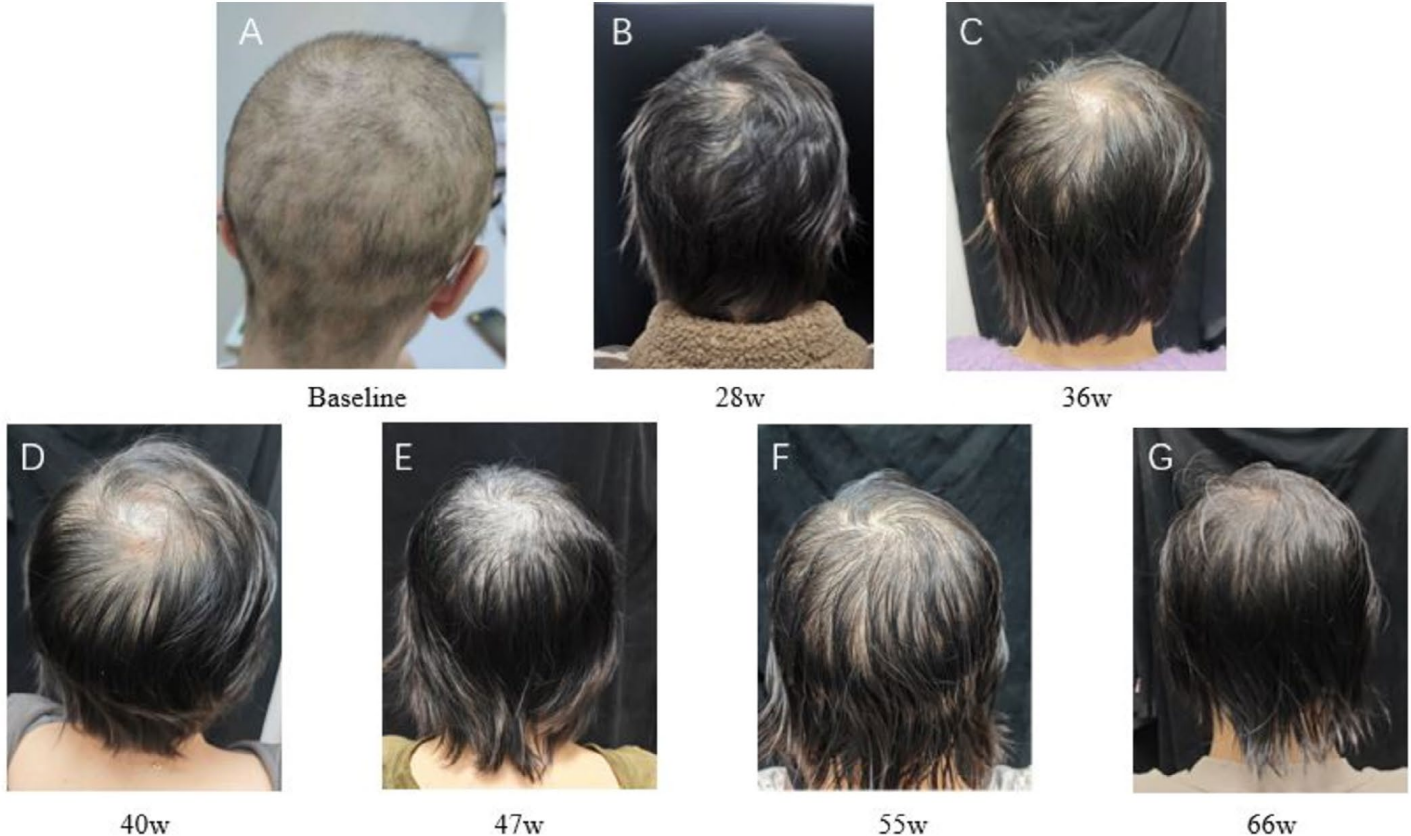
Imaging documentation from baseline to 66 weeks.

In June 2023, she visited our dermatology department. A skin examination revealed sparse scalp hair (Figure 1A). The Severe Alopecia Tool (SALT) score was used to assess her hair loss, yielding a score of 80, indicating severe alopecia. Comprehensive baseline assessments, including complete blood counts, liver and kidney function tests, thyroid hormone levels, serum IgE, vitamin D, trace elements, and tuberculosis screening (T‐SPOT), were all within normal limits. Based on her clinical presentation and medical history, she was diagnosed with alopecia universalis. After obtaining written informed consent, she initiated treatment with tofacitinib 5 mg twice daily.

At the 28‐week follow‐up (Figure 1B), her SALT score decreased to 5, and there was a noticeable increase in hair density. Blood routine and liver and kidney function tests remained stable throughout the treatment period, with no systemic side effects observed, allowing for continued tofacitinib therapy. However, at the 36‐week follow‐up (Figure 1C), her SALT score increased to 12, and hair loss resumed, with a positive hair‐pull test. Despite this, the patient declined switching medications and opted for corticosteroid injections to prevent further hair loss. At 36 weeks, she received an intramuscular injection of betamethasone.

At the 40‐week follow‐up (Figure 1D), significant hair loss persisted, with a SALT score of 30 and a positive hair‐pull test. Another 5 mg intramuscular betamethasone injection was administered, and tofacitinib treatment was continued. By 47 weeks (Figure 1E), hair loss had largely ceased, the hair‐pull test was negative, and her SALT score was 5. Tofacitinib treatment was continued. However, at the 55‐week follow‐up (Figure 1F), her SALT score rose to 10. After a thorough discussion with the patient, we decided to switch to ritlecitinib for subsequent treatment. Encouragingly, at the 66‐week follow‐up (Figure 1G), her SALT score was 5, and the hair‐pull test remained negative, indicating improvement compared to the 55‐week follow‐up. We continue to monitor her condition closely. Unfortunately, during the most recent follow‐up visit, no significant signs of hair regrowth were observed.

Throughout the entire treatment process, we strictly monitored the patient's complete blood cell count (CBC), liver and kidney functions, as well as D‐dimer levels. All indicators remained within the normal range. No adverse events, including herpes virus infection, folliculitis, or headache, were observed. The patient only occasionally suffered from upper respiratory tract infections (Table 1).

**TABLE 1:** Patient dose adjustment timeline.

Treatment timepoint (weeks)	Interventions	Dosage/usage	SALT score	Adjustment reason
Baseline	Tofacitinib	5 mg, twice daily	80	/
28	Tofacitinib	5 mg, twice daily	5↓	/
36	Tofacitinib + Betamethasone	5 mg, twice daily + 5 mg, single injection	12↑	Combined treatment with corticosteroids during the acute hair loss period to prevent the expansion of the hair loss area.
40	Tofacitinib + Betamethasone	5 mg, twice daily + 5 mg, single injection	30↑	Combined treatment with corticosteroids during the acute hair loss period to prevent the expansion of the hair loss area.
47	Tofacitinib	5 mg, twice daily	5↓	/
55	Ritlecitinib	50 mg, once daily	10↑	The hair loss score has significantly increased.
66	Ritlecitinib	50 mg, once daily	5↓	/

*Note:* The positive criterion for the Hair‐pull Test: > 6 hairs per pull. ↑↓ represents an increase or decrease in the SALT score compared to the previous follow‐up. Betamethasone was an adjunctive treatment and did not substitute for the JAK inhibitors (Tofacitinib/Ritlecitinib). During the entire disease course, blood routine, liver and kidney functions, and D‐dimer were all normal, with only occasional upper respiratory tract infections.

## 3 Discussion

AA is a condition that affects hair follicles, leading to non‐scarring hair loss. The complex pathogenic mechanisms of AA are not yet fully understood. Recent studies indicate that the disruption of immune privilege in hair follicles results in the accumulation of inflammatory mediators and cells at the follicular site, leading to follicular damage and subsequent hair loss. Activated CD8+ NKG2D+ T cells play a pivotal role in the pathophysiology of AA. These cells activate and secrete substantial amounts of IFN‐γ via the JAK1 and JAK3 signaling pathways. IFN‐γ not only induces a pro‐inflammatory response but also stimulates IL‐15 production in follicular epithelial cells through the JAK1 and JAK2 pathways. The interplay between IL‐15 and CD8+ NKG2D+ T cells creates a reinforced positive feedback loop that perpetually enhances the inflammatory response [1, 3]. Recent studies have demonstrated that, besides the conventional TH1 route, immunological regulatory mechanisms like TH2, TH9/IL‐9, TH17, and IL‐23 contribute to the pathogenesis of AA [3].

In the field of treatment, JAK inhibitors have demonstrated promising prospects. By blocking cytokine signaling pathways closely associated with autoimmune responses, they effectively suppress immune attacks on hair follicles, thereby promoting hair regrowth. Further research has revealed that the role of JAK inhibitors extends beyond reducing immune attacks; they can also activate and promote the proliferation of hair follicle stem cells in the growth phase by upregulating the Wnt (hair follicle induction) and Shh (morphogenesis and late differentiation) signaling pathways, thus prolonging and accelerating the hair regrowth process [8].

In vitro experiments have shown that although Ruxolitinib alone does not directly stimulate the expression of key molecules such as Wnt7, Lef1, and DKK1, it can effectively reverse the transcriptional changes in the Wnt/β‐catenin signaling pathway induced by IFN‐γ in human dermal papilla cells (hDPCs) [9]. Ruxolitinib activates critical molecules in the Wnt/β‐catenin pathway, including Lef1 and β‐catenin, and inhibits DKK1 expression. Additionally, Ruxolitinib stimulates the expression of various growth factors, such as FGF7. The upregulation of FGF7 in hDPCs regulates BMP signaling (a member of the TGF‐β superfamily, which plays a crucial role in embryonic development, bone formation, and tissue homeostasis by regulating cell proliferation, differentiation, and apoptosis), promoting hair follicles to enter the anagen phase and extending its duration [9]. Ruxolitinib treatment also reduces the expression of MHC II molecules in the hair bulb and surrounding areas, preventing the collapse of hair follicle immune privilege induced by IFN‐γ. Harel et al. reported that tofacitinib treatment during the mid‐anagen phase in mice promoted hair growth, possibly by increasing the expression of TGF‐β2, BMP6, and Lef1 in human DP spheres [8]. Moreover, JAK inhibitors may promote angiogenesis by inducing VEGF overexpression, providing essential nutritional support for hair follicles and further enhancing hair regrowth [10]. These findings indicate that JAK inhibitors have multiple potential mechanisms in hair regrowth therapy, offering broad possibilities for future research and clinical applications.

The determination of reliable biomarkers for evaluating the efficacy of JAK inhibitors in treating AA requires multi‐dimensional analysis. From the perspective of JAK inhibitors' inhibition of the JAK‐SATA signaling pathway, changes in T‐cell‐related inflammatory markers (IFN‐γ, IL‐17, and CXCL10) in scalp biopsies reflect the degree of suppression of autoimmune attacks. Regarding other possible mechanisms of action, molecular biomarkers in the Wnt/β‐catenin pathway show particular predictive value: the upregulation of Lef1 and β‐catenin after treatment may indicate better hair regrowth outcomes. Monitoring angiogenic indicators (serum VEGF levels and microvascular density around hair follicles) may reveal dynamic changes corresponding to the treatment stage.

The mechanism of relapse following effective treatment with Tofacitinib remains ambiguous and may be associated with deficiencies in the quantity and functionality of regulatory T cells (Tregs). Tregs play a crucial role in suppressing the functions of CD8+ T cells, natural killer (NK) cells, and CD4+ T cells through direct cell contact or the release of soluble factors such as TGF‐β and IL‐10. Additionally, Tregs can express the Notch ligand Jag1, which is recognized for its role in stimulating hair growth. In a Th2‐dominant environment, Tregs may adopt a Th2‐like phenotype, leading to reduced quantity and functionality, which can facilitate the recurrence of AA [11]. A further potential explanation for recurrence following AA treatment is the presence of a minor population of pathogenic tissue‐resident memory T (TRM) cells in the dermis. TRM cells are long‐lived lymphocytes located in tissues that emerge after the onset of T cell‐mediated immune responses, functioning to ensure tissue surveillance and eliminate infections in conjunction with other effector and TRM cell populations. While JAK inhibitors can eradicate some cells, the residual small population of pathogenic TRM cells in the skin may promote disease recurrence. Pathogenic tissue‐resident TRM cells have been implicated in several autoimmune disorders, including psoriasis, vitiligo, atopic dermatitis, and cutaneous lupus erythematosus [12, 13]. Consequently, if the activity of pathogenic TRM cells can be successfully suppressed and eradicated from the affected dermis, it may lead to prolonged disease remission in AA.

Although treatment drugs like Tofacitinib effectively stimulate rapid hair regrowth in AA patients during the initial phase, the complex pathological mechanisms involved, including multifaceted immune and genetic regulatory pathways, present a risk of potential recurrence during therapy. For individuals exhibiting reduced efficacy during JAK inhibitor therapy, it is crucial to proactively investigate and modify treatment strategies during the active disease phase.

### 3.1 Strategy 1: Combined Drug Therapy

Corticosteroids have long served as a primary treatment for AA, particularly during acute phases of the condition. Research comparing TNF‐α levels in the serum and tissue of AA patients before and after corticosteroid therapy revealed elevated levels compared to the control group both pre‐ and post‐treatment, with a significant reduction observed after treatment [14]. It has been reported that patients who fail to respond to tofacitinib monotherapy may exhibit a response to the combination therapy of tofacitinib and prednisone [6]. While studies suggest that oral tofacitinib in conjunction with corticosteroids may offer greater efficacy, reduced dosing requirements, and fewer adverse reactions compared with corticosteroid monotherapy [15], conclusive evidence delineating the differences between tofacitinib monotherapy and corticosteroid combination therapy remains limited. Although corticosteroids demonstrate promising efficacy in severe AA, their long‐term use is restricted due to a high incidence of adverse events.

Moreover, investigating alternative combination therapies may prove advantageous. Minoxidil, a common topical medication in dermatology, stimulates hair growth through multiple mechanisms, including vasodilation, opening potassium (K+) channels, and activating extracellular signal‐regulated kinase (ERK) and protein kinase B (AKT/PKB) signaling pathways. Additionally, minoxidil has been shown to reduce the resting phase duration, extend the anagen phase, enhance follicle size, and promote follicle proliferation and differentiation [16, 17]. Researchers like Carlos G. have proposed that combining Tofacitinib with oral minoxidil may produce better outcomes than Tofacitinib alone. One patient who had previously received no benefit from Tofacitinib treatment (11 mg once daily for 3 months) exhibited significant hair regrowth during the initial 3 months of combined therapy with the same dosage of Tofacitinib (5 mg twice daily) [18]. Furthermore, co‐administration of oral minoxidil may demonstrate therapeutic efficacy after relapse, suggesting that combined oral minoxidil could be a viable alternative therapy.

However, the therapeutic efficacy of Tofacitinib in AA is not consistently effective, potentially due to Th2 skewing, which reduces both the quantity and functionality of Tregs [11]. Tregs are known to enhance the proliferation and differentiation of hair follicle stem cells, thereby facilitating hair growth [19]. In the context of AA, Treg levels are diminished, and IL‐2 has been shown to promote Treg proliferation in AA patients [20]. Consequently, integrating low‐dose IL‐2 into the treatment protocol alongside Tofacitinib may alleviate hair loss. For AA patients unresponsive to Tofacitinib, the addition of low‐dose IL‐2 therapy may represent a promising approach.

The patient initially exhibited a favorable response to Tofacitinib monotherapy; however, secondary failure subsequently developed. After consultations with the patient, it was decided to initiate corticosteroid therapy during the acute phase, which successfully mitigated the symptoms of hair loss. However, the potential adverse effects associated with corticosteroids render long‐term use impractical. Following discontinuation of corticosteroids, the patient showed no significant improvement in hair growth, indicating the need for a modification in the treatment plan.

### 3.2 Strategy 2: Increase the Dose of Tofacitinib

The dosage and duration of oral Tofacitinib therapy can significantly influence its effectiveness in managing AA. Patients administered twice‐daily doses exceeding 5 mg of Tofacitinib for more than 6 months exhibited significantly superior outcomes compared to those receiving doses of 5 mg or less for 6 months or fewer [21]. Two significant studies, BRAVE‐AA1 and BRAVE‐AA2, involving 1200 adult patients with severe AA, highlighted the potential benefits of modifying treatment regimens. Patients who did not respond to 2 mg of Baricitinib at week 52 showed substantial regrowth of hair, eyebrows, and eyelashes by week 76 after dose adjustments [22]. Consequently, for patients experiencing a relapse of AA during Tofacitinib therapy, extending the treatment duration or increasing the dosage may be a viable strategy to enhance therapeutic efficacy. In the case report examined, the patient declined the suggestion to increase the Tofacitinib dosage, potentially due to concerns about adverse effects or other personal considerations.

### 3.3 Strategy 3: Switch to Other JAK Inhibitors

Patients who relapse following an initial positive response to oral Tofacitinib for alopecia areata (AA) or those who exhibit a poor response at therapy onset may benefit from transitioning to alternative JAK inhibitors [23]. This theory was demonstrated in a case study of a female patient with a 30‐year history of AA. After ineffective trials with Tofacitinib (30 mg twice daily) and dupilumab, she switched to ruxolitinib and achieved full regrowth of her scalp, eyebrows, and eyelashes within 8 months [24]. It is crucial to recognize that Tofacitinib functions as a JAK1/JAK3 (>JAK2) inhibitor, while ruxolitinib serves as a JAK1/2 inhibitor. Despite their overlapping selectivities, these two JAK inhibitors exhibit significantly differing efficacies in the same patient. Regrettably, the scientific community has not yet reached a consensus on the underlying reasons for the substantial disparities in efficacy reported among different JAK inhibitors. A potential explanation is that a particular JAK inhibitor, besides its direct effect on its target, may also indirectly affect other kinome members. This intricate network of connections ultimately determines the therapeutic outcome for the patient.

Ritlecitinib is another available JAK inhibitor. Phase 2a randomized, double‐blind, placebo‐controlled clinical research has shown that Ritlecitinib significantly decreases TH1 marker expression while enhancing KRT expression in the scalp of patients with AA [25]. In addition to its function in the JAK–STAT signaling pathway, Ritlecitinib may also indirectly affect IFN‐γ production by blocking TEC family kinases [26]. When certain JAK inhibitors fail to produce the intended therapeutic outcome—either due to a lack of initial response or relapse following effective treatment—transitioning to a different JAK inhibitor is a feasible approach.

**FIGURE 2:**
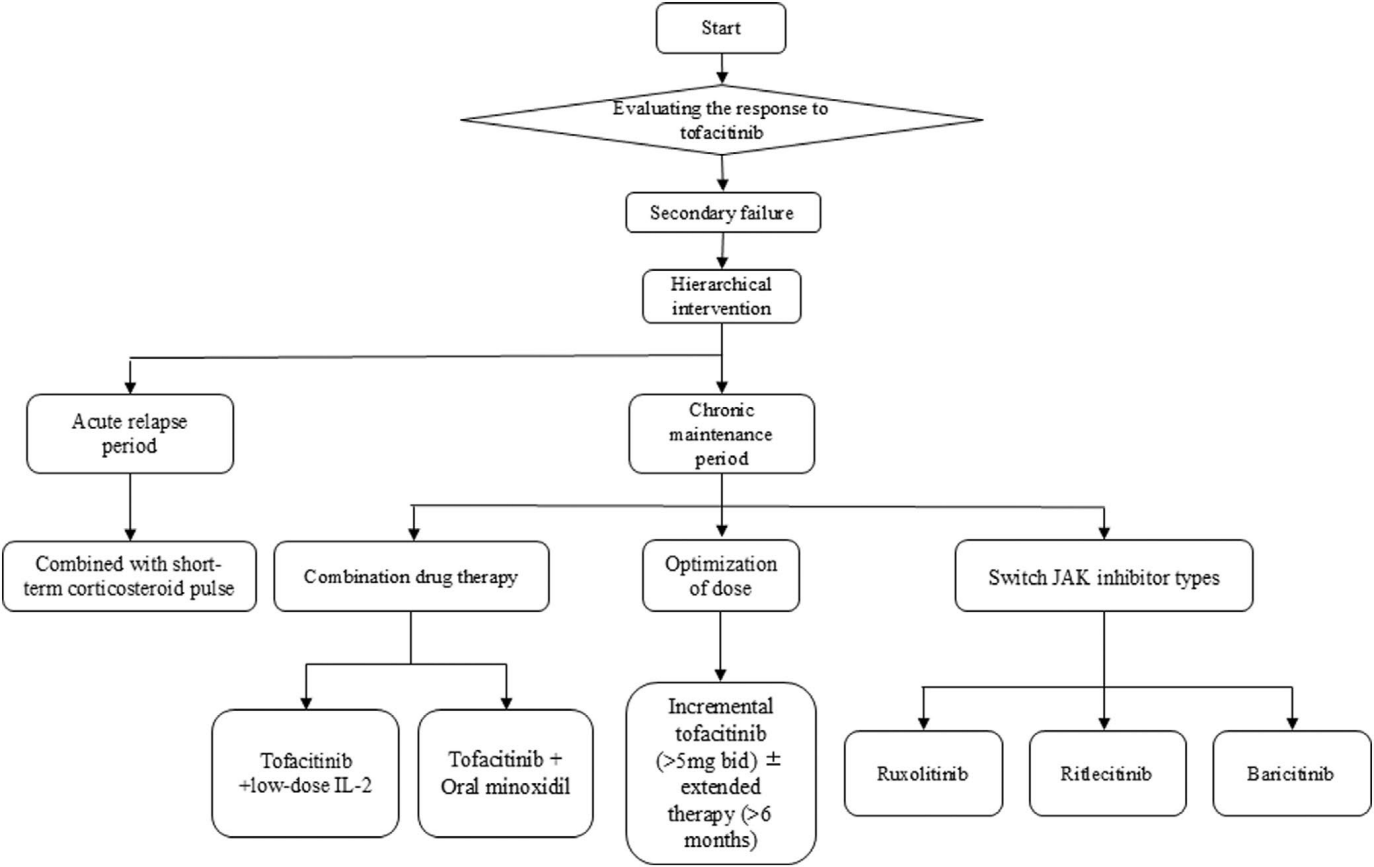
Clinical decision‐making algorithm. Secondary failure: In instances where the initial treatment is effective (i.e., a satisfactory primary response is achieved), over time, the therapeutic efficacy progressively diminishes, ultimately resulting in disease recurrence or progression.

Although JAK inhibitors have shown great potential in reversing AA immune‐mediated alopecia, they also carry the potential risk of causing serious side effects. The black box warning issued by the US Food and Drug Administration (FDA) for JAK inhibitors highlights risks including serious cardiovascular events, thrombosis, malignancies, and severe infections. Analyses of adverse events associated with ruxolitinib, tofacitinib, and baricitinib indicate that JAK inhibitors are most frequently linked to infectious adverse events, embolism and thrombosis, tumors, and gastrointestinal perforation [27]. Compared with the control group (receiving adalimumab or etanercept), patients with rheumatoid arthritis treated with 5 or 10 mg tofacitinib twice daily had a higher incidence of major adverse cardiovascular events (MACE) and cancer [28]. In contrast, no increased risk of MACE or cancer was observed in the overall population of ulcerative colitis patients treated with tofacitinib [28]. The differences in MACE and cancer risk between rheumatoid arthritis and ulcerative colitis patients suggest that the risks associated with JAK inhibitors may vary across different patient populations, necessitating further randomized studies to clarify these discrepancies. Prior to initiating treatment, screening for tuberculosis (T‐SPOT/PPD), hepatitis B/C, HIV, latent fungal infections, and tumor markers should be conducted. During treatment, regular monitoring of blood routine, D‐dimer levels, tumor markers, and liver and kidney function is essential, along with close observation of symptoms and signs. If persistent fever, cough, skin ulcers, or other signs of infection, suspected thrombosis symptoms (leg swelling, chest pain, shortness of breath), or unexplained weight loss and lymph node enlargement occur, medication should be stopped immediately, and appropriate symptomatic treatment provided (Figure 2).

## 4 Conclusion

JAK inhibitors have ushered in a new era of optimism for the treatment of AA. Although the JAK–STAT signaling pathway plays a pivotal role in the pathophysiology of AA, it is likely not the sole mechanism involved. Given the significant inter‐patient variability and the complex nature of AA's pathophysiology, treatment outcomes can differ markedly. When monotherapy proves ineffective, alternative treatment strategies should be considered.

## Author Contributions

The authors confirm contribution to the paper as follows: study conception and design: Yao longyan, He Jiume, Lan na; analysis and interpretation of results: Yao longyan, He Jiume, Lv Yongmei; draft manuscript preparation: Yao longyan, He Jiume, Lv Yongmei. All authors reviewed the results and approved the final version of the manuscript.

## Ethics Statement

This study is approved by the Ethics Committee of the Second Affiliated Hospital of Anhui Medical University. Approval code is YX2024‐154.

## Consent

Written informed consent was obtained from the patient for the publication of all the images and data included in this article.

## Conflicts of Interest

The authors declare no conflicts of interest.

## Data Availability

The data that support the findings of this study are available from the corresponding author upon reasonable request.
